# Primary Aspergillosis of the Larynx

**DOI:** 10.1155/2016/1234196

**Published:** 2016-02-03

**Authors:** Richard H. Law, Samuel A. Reyes

**Affiliations:** Department of Otolaryngology, University at Buffalo School of Medicine and Biomedical Sciences (SUNY), 1237 Delaware Avenue, Buffalo, NY 14209, USA

## Abstract

Laryngeal aspergillosis is most commonly seen as a result of secondary invasion from the lungs and tracheobronchial tree in immunocompromised hosts. Primary aspergillosis of the larynx is, however, rare with few cases documented over the past fifty years. We report a case of a 73-year-old woman who presented with persistent hoarseness. She is a nonsmoker with a history of asthma and chronic bronchiectasis treated with bronchodilators, inhaled and oral corticosteroids, and nebulized tobramycin. Direct laryngoscopy with vocal cord stripping confirmed the diagnosis of invasive aspergillosis with no manifestations elsewhere. The patient was successfully treated with oral voriconazole with no signs of recurrence. Although several major risk factors contributing to the development of primary aspergillosis of the larynx have been discussed in the literature, there has been no mention of inhaled antibiotics causing this rare presentation to the best of our knowledge. We, therefore, highlight the use of inhaled tobramycin as a unique catalyst leading to the rapid onset of this rare presentation.

## 1. Introduction

Disseminated invasive aspergillosis is most commonly associated with immunocompromised states such as AIDS, malignancies, aplastic anemia, chemotherapy, radiation, immunosuppressants, and genetic disorders of the immune system [1].* Aspergillus* has very little pathogenic capability in a healthy host; however, it can cause major morbidity and mortality in compromised hosts [2, 3]. It gains access to the respiratory mucosa via inhalation (spores) with subsequent invasion causing necrosis, ulceration, hemorrhage, and thrombosis. In immunocompromised hosts, there is often hematogenous seeding involving multiple organs such as the lungs, brain, heart, kidneys, spleen, gastrointestinal tract, and lymph nodes [4–6].

Despite its opportunistic nature,* Aspergillus* can also cause localized/primary disease in relatively healthy patients with the external auditory canal, paranasal sinuses, and orbit being the most common sites affected [2, 4, 5, 7]. Primary aspergillosis of the larynx is, however, extremely rare when compared to the incidence of primary aspergillosis affecting other sites within the head and neck. As a result, this unique presentation may be mistaken for malignancy of the vocal folds initially. The exact mechanism of primary aspergillosis of the larynx is still unclear but is most likely multifactorial.

## 2. Case Report

The patient is a 73-year-old woman who was referred by her pulmonologist for persistent hoarseness, which began after starting nebulized tobramycin nine months earlier. She is a nonsmoker with a history of asthma and chronic bronchiectasis treated with inhaled ipratropium bromide/albuterol, oral prednisone, and an inhaled combination of budesonide and formoterol, prior to starting nebulized tobramycin. She did not have any malignancies or other known active infections. CRP, ESR, CBC with differential, serum immunoglobulins, and* Aspergillus* immunoglobulins were negative. On exam, there were no visible lesions or masses in the oral cavity as well as the oropharynx and nasopharyngeal mucosa. There was no evidence of cervical lymphadenopathy or palpable masses in the neck. Her voice was noted to be hoarse with a whisper-like quality.

**Figure 1:**
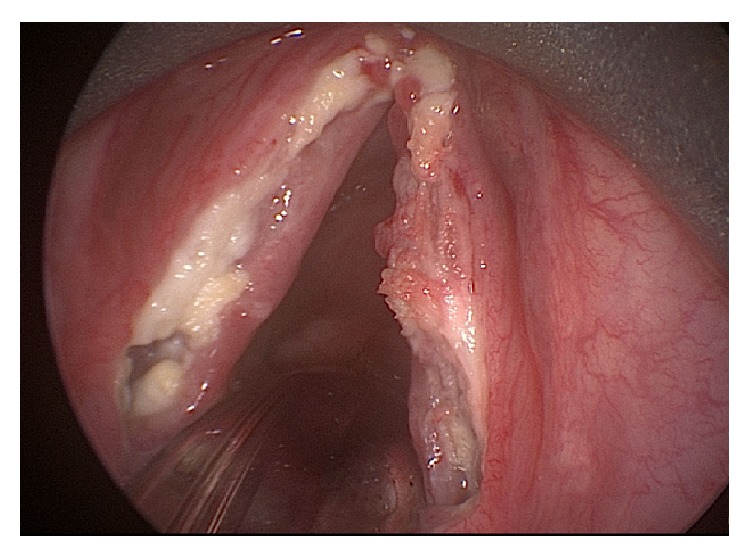
Invasive aspergillosis of the larynx.

**Figure 2:**
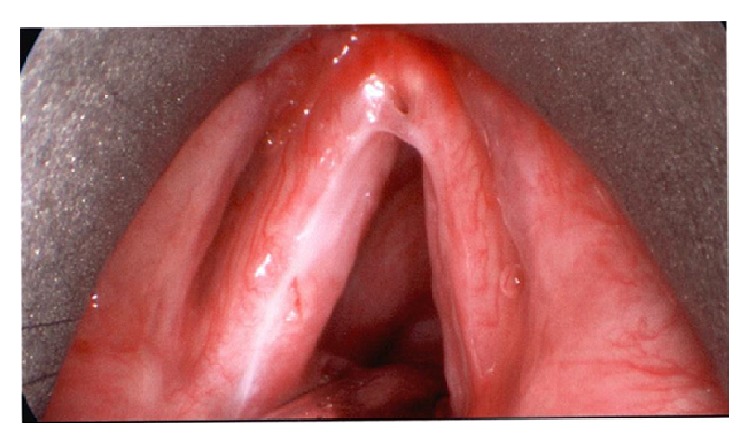
Resolution of invasive aspergillosis.

Laryngoscopy revealed extensive leukoplakia and inflammation of the true vocal folds bilaterally (Figure 1). Both true vocal cords were noted to have full mobility. Vocal cord stripping was performed, and the pathological analysis revealed necrosis with invasive fungus. There were no signs of malignancy, and the fungal morphology was consistent with* Aspergillus* species. CT with contrast also showed no fungal disease elsewhere in the respiratory tract. The patient was subsequently treated with oral voriconazole for five months. During the course of her treatment, her inhaled corticosteroids were discontinued, and she was able to be weaned off the bronchodilators. In addition, her oral prednisone dose was slowly weaned over the course of five months and eventually discontinued. Over this time she demonstrated gradual improvement in her hoarseness and resolution of the leukoplakia on laryngoscopy; there was some webbing noted near the anterior commissure (Figure 2). Repeat biopsy with culture found no further infection or inflammation. The only notable acute event that occurred during her treatment course was the development of a lower extremity DVT; she was treated in the hospital and started on Xarelto.

## 3. Discussion

The incidence of primary aspergillosis of the larynx is still exceedingly rare with few cases documented over the past fifty years [3, 5, 8]. Although the exact pathogenesis of this rare disease is not entirely clear, different risk factors for the development of primary aspergillosis of the larynx have been extensively discussed. Inhaled tobramycin, however, has never been mentioned as a factor contributing to this rare disease to date.

There are several common predisposing factors leading to primary aspergillosis of the larynx that are frequently found in the literature. They include inhaled corticosteroids for chronic respiratory diseases, vocal cord abuse, smoking, severe reflux disease, laryngeal radiation, and settings of prolonged exposure to large amounts of fungal spores [3, 7, 9–14]. These factors cause either decrease in local immunity, direct damage to the protective mucosal barrier, or increase in exposure to* Aspergillus*. Any one factor alone is probably not enough to cause primary aspergillosis of the larynx [4–6]. Rather, a combination of the host and environmental factors most likely contributes to the development of this unique disease. Also, some risk factors play a stronger role than others.

Our patient had two major risk factors leading to the development of primary aspergillosis: corticosteroids (systemic and inhaled) and nebulized tobramycin. There is currently no mention of nebulized antibiotics contributing to the development of primary aspergillosis of the larynx in the literature. Only Nong et al. and Ran et al. described patients most similar to our patient [12, 15]. These patients were also on combination corticosteroid therapy; however, they were on systemic antibiotics instead of inhaled antibiotic. Prolonged use of systemic antibiotics alters the dynamics of the local flora of the larynx allowing fungal colonization [4, 12, 15]. We suspect that inhaled antibiotics also alter the flora of the laryngeal mucosa, but with an amplified local effect; this can be supported by the rapid onset of hoarseness in our patient.

The timeline on which our patient developed symptoms is thus particularly notable. Despite long-term corticosteroid use, our patient never developed hoarseness or increased work of breathing. She only developed hoarseness shortly after initiating nebulized tobramycin. This contrasts with other patients in the literature where they developed symptoms after being on corticosteroids and systemic antibiotic therapy for many years. Furthermore, our patient was on a nebulized antibiotic monotherapy compared to the combination systemic oral antibiotic therapy described in the other cases [4, 12, 15]. This observation also supports that nebulized antibiotics can have stronger local influences leading to a more rapid development of laryngeal aspergillosis than oral antibiotics.

The exact etiology of this rare disease remains elusive but is most likely multifactorial with a complex interplay between host and the environmental factors. The severity of the disease is also highly variable and dependent on the synergistic effects of various combinations of risk factors. Treatment is thus tailored for the severity of the disease. By presenting this patient, we introduce a new risk factor/etiology for primary aspergillosis of the larynx and broaden the context in which this rare disease may present.
